# Globalization and the health and well-being of migrant domestic workers in Malaysia

**DOI:** 10.1186/s12992-023-00925-w

**Published:** 2023-04-24

**Authors:** Denise L. Spitzer, Shanthi Thambiah, Yut Lin Wong, Manimaran Krishnan Kaundan

**Affiliations:** 1 School of Public Health, University of Alberta, Edmonton, Canada; 2 Institute of Feminist and Gender Studies, University of Ottawa, Ottawa, Canada; 3 Gender Studies Programm, Faculty of Arts and Social Sciences, University of Malaya, Kuala Lumpur, Malaysia; 4 Social & Preventative Medicine Department, Faculty of Medicine, University of Malaya, Kuala Lumpur, Malaysia; 5 Institute for Health Behavioural Research, National Institutes of Health, Ministry of Health, Shah Alam, Malaysia

**Keywords:** Migrant domestic workers, Malaysia, Indonesia, Philippines, Gender, Health, Well-being, Labour

## Abstract

**Background:**

Neoliberal globalization contributes to the out-migration of labour from sending countries in the global South. Supported by multilateral organizations including the IMF and World Bank, the migration and development nexus holds that nations and households in migrant sending countries can migrate their way out of poverty. Two countries that embrace this paradigm, the Philippines and Indonesia, are major suppliers of migrant labour including domestic workers, and Malaysia is a primary destination country.

**Theory and methods:**

We deployed a multi-scalar and intersectional lens to highlight the impact of global forces and policies, interacting with constructions of gender and national identity, to explore the health and wellbeing of migrant domestic workers in Malaysia. In addition to documentary analysis, we conducted face-to-face interviews with 30 Indonesian and 24 Filipino migrant domestic workers, five representatives from civil society organizations, three government representatives, and four individuals engaged in labour brokerage and the health screening of migrant workers in Kuala Lumpur.

**Results:**

Migrant domestic workers in Malaysia work long hours in private homes and are not protected by labour laws. Workers were generally satisfied with their access to health services; however, their intersectional status, which is both an outcome of, and contextualized by, the lack of opportunities in their own country, prolonged familial separation, low wages, and lack of control in the workplace, contributed to stress and related disorders—which we regard as the embodied manifestation of their migratory experiences. Migrant domestic workers eased these ill effects through self-care, spiritual practices, and the embrace of gendered values of self-sacrifice for the family as a form of solace.

**Conclusions:**

Structural inequities and the mobilization of gendered values of self-abnegation underpin the migration of domestic workers as a development strategy. While individual self-care practices were used to cope with the hardships of their work and family separation, these efforts did not remedy the harms nor redress structural inequities wrought by neoliberal globalization. Improvements in the long-term health and wellbeing of Indonesian and Filipino migrant domestic workers in Malaysia cannot focus solely on the preparation and maintenance of healthy bodies for productive labour, but must attend to workers’ attainment of adequate social determinants of health, which challenges the migration as development paradigm. Neo-liberal policy instruments such as privatization, marketisation and commercialization of migrant labour have led to both host and home countries benefitting, but at the expense of the migrant domestic workers’ well-being.

## Introduction

Neoliberal globalization is characterized by reduced public support for services, heightened socioeconomic inequities, the burgeoning of precarious labour, and exacerbated environmental degradation contributing to pressures, discourses, and an elaborate infrastructure that supports the out-migration of labour from sending countries in the global South [1]. Concomitantly, increased female labour market participation in middle- and upper-income countries has generated gaps in care-work and domestic labour responsibilities that are regarded as the purview primarily of women in the private sphere—gaps now being filled by women migrant workers from less affluent countries [2, 3]. The movement of migrant domestic care workers involves transit across national borders, which paves the way for blurring the boundaries between private and public spheres, inveigling households, private industry, and governments in the placement and control over migrant women’s bodies [4].

In this paper, we employ a multi-scalar and intersectional lens that attends to the mutually constituted phenomena of gender, migration and socioeconomic status, which includes ethnicity, religion, and other social markers, that situate migrant domestic workers in Malaysia within the social landscape of the globalization of care work and its consequent influence on their health and wellbeing. Although health services are made available to these migrant workers, which may address health issues in the short-term, the globalization of care work and various forms of discrimination intersect with each other and create overlapping and reinforcing barriers producing heighten vulnerabilities. These intersectionalities limit their access to social determinants of health that may have more embodied long-term effects on their health and well-being.

## Context

The increasing prominence of migrant domestic workers encompasses four major migration trends: the rise in transnational linkages, the increased importance of labour migration, the prominence of women and the increase in undocumented workers [5]. Notably, domestic labour is inextricably linked with female gender roles throughout much of the world [6] and domestic work is the “single largest occupation excluded from labour and social protection” [2, p.15]. As women enter the work force, women from poorer countries are hired as migrant workers to take on their domestic roles thus creating a ‘global care chain’ [7, 8].

### The Philippines and Indonesia: deployment of migrant domestic workers

The Philippines remains the world’s primary source of migrant domestic workers [9, 10]; however, Indonesia has also become a major supplier of migrant labour to the world [11]. Both countries have developed sophisticated infrastructure that engages multi-scalar (village, national, transnational) public and private sector actors working in concert with policies, services, and supportive discourses promoted by governments and multilateral organizations such as the International Monetary Fund, the World Bank, among others, to facilitate the out-migration of their nationals thereby shifting the responsibility of lifting their families and their home country out of poverty to migrant workers [1, 10–13].

Indonesia has been emulating the Philippine model, underscoring that their workers, generally less educated and reputedly more “docile” than their Philippine counterparts, can be hired at lower wages [10, 11, 14]. Once Indonesian women agree to be recruited as migrant labourers, they are relocated to a training centre where they are confined and isolated from contact with the outside world until they are dispatched for overseas placement [11, 12, 15]. The Indonesian workers are indebted to the recruitment agency upon arrival at the training centre for their training and placement fees; these monies are to be reimbursed through levies placed on their salaries for an average of seven to nine months [11, 15]. To mitigate against the possibility that workers may back-out or run away from the training centre, family property (land, vehicles, etc.) is often secured as collateral [12]. By contrast, in the Philippines, prospective migrants must pay numerous fees upon signing a contract with a recruitment agency, monies that may be secured from kin or local loan agents. Once the funds are received and the contract is signed, the prospective migrant worker is at liberty to reside anywhere until the work visa has been received and overseas deployment is imminent [15].

Migrant domestic workers are often socially as well as financially indebted. Constructed as carers for their families and the nation, they are tasked with contributing to their financial as well as social wellbeing. Consequently, women migrants are morally required to fulfil their roles as filial daughters, wives, sisters, and mothers by working hard and sending remittances back to their families [1, 12, 16]. Where care labour has become a prominent national export, millions of women have left their countries to enter foreign domestic work, leaving children in the hands of extended family—often for years at a time [1, 9, 17]. While children and their families left behind may benefit from remittances; the social and personal cost of familial separation is also significant [18, 19].

### Migrant domestic workers in Malaysia

Malaysia has been reliant on migrant labour to sustain and expand its economy since colonial times [20]. Around the time of our data collection, in addition to thousands of other migrant labourers working in other sectors, the Ministry of Home Affairs Malaysia reported as of 30 June 2019 there were 129,168 registered domestic workers —72% of whom were from Indonesia (92,405) and 25% from the Philippines (32,277), and the remainder from other South and Southeast Asian countries [21, p.37]. The International Labour Organization (ILO), however, estimated that Malaysia hosts between 300,000 and 400,000 migrant domestic workers [22, p.21].

Although Malaysia is signatory to the Convention on the Elimination of All Forms of Discrimination (CEDAW), it has not yet ratified the International Convention on the Protection of the Rights of All Migrant Workers and Members of Their Families [14, 23]. Moreover, migrant domestic workers are not covered by Malaysian labour laws such as the Employment Act of 1955 and the Workmen’s Compensation Act of 1952 as they are ostensibly regarded not as workers, but as domestic helpers engaged in the informal labour sector who are discursively constructed as family members [14, 16, 24–26]. According to a global survey, domestic workers in Malaysia report the world’s longest work week for this occupational category averaging 65.9 h [16]. Only 11% recount working eight hours a day or less while the workday for 46% extends 15 h or more; 42% describe working seven days a week [21].

Migrant domestic workers are required to reside with their employers to whom they are tied for a two-year period [16, 26]. Although Filipino migrant workers receive higher salaries than their Indonesian counterparts [16], complaints have been raised about contract substitution amongst Filipino migrant workers where their contract outlining salary, rights, and duties signed in English in the Philippines is replaced with a document in Bahasa Malaysia that stipulates different conditions [26]. Many employers restrict the movement of their employees and circumscribe their interactions with both co-nationals and local citizens to limit opportunities for both critique of their situations and potential romantic entanglements [4, 16, 20, 26].

Lack of labour protection and the private nature of their work, makes them vulnerable to abuse [14, 20, 27]. These threats are exacerbated by the fact that while migrant domestic workers are supposed to be granted a rest day per week, not all employers comply with this regulation [26]. When the rest day was mandated for all domestic workers, employers were highly resistant to these conditions as some employers’ fear that their workers may not return [11, 28, 29]. Despite their importance in supporting the Malaysian women’s labour force participation, migrant domestic workers, like other migrant workers, are often portrayed in the media as potential criminals or as depraved individuals [4, 29]. These stereotypes persist despite evidence that a very small percent of all crimes is committed by foreign workers in peninsular Malaysia [30].

Salaries were also left to the determination of the market forces [14]. Indonesian domestic workers, receive disparate treatment including lower wages and until recently, fewer or no days off as compared to Filipina domestic workers who benefit from a standard contract implemented with the support of the Philippine government. Indonesia issued a moratorium on sending domestic workers to Malaysia in 2009, reducing the numbers of Indonesian migrant domestic workers from an average of 1,000 per month to 200 in January 2011^1^ [31]. Another agreement issued in 2011 guaranteed a rest day for Indonesian migrant domestic workers and allowed workers to retain their passports, which previously had been held by employers; however, no minimum salaries were instituted at that time [27, 31].^2^ A Memorandum of Understanding (MOU) between Indonesia and Malaysia signed in 2006 required employers to sign a standard contract; however, workers were still required to reside with their employer for the duration of their two-year contract [11]. Moreover, the MOU and subsequent 2011 Amending Protocol have not made much contribution to migrant domestic worker protections [32, 33].

### Migration and health

Migrant workers, with the notable exception of managerial level expatriates, are compelled to undergo a complete health examination prior to qualifying for a work permit [23, 34]. Only those deemed free of any communicable disease, serious illness or pregnancy, are declared fit to enter the country. Thereafter, workers must follow up with an additional medical exam within a month of arrival and are subject to annual health examinations administered by a private agency on contract with the government, the Foreign Workers Medical Examination Agency (FOMEMA), to determine continued fitness to remain in the country or be subject to deportation [23, 34].

Access to health care services in Malaysia’s mixed public and private system can be fraught for migrant workers [23, 35]. Despite mandatory insurance schemes, most migrant workers will attend public health facilities where they pay a fee before receiving treatment, except in emergencies when payment is sought afterwards. In addition to the economic barrier, patients are required to show evidence of their legal status and some employers still retain control over workers’ passports making it difficult to assert their right to care.

Health is determined by much more than the presence or absence of disease but is instead influenced by a complex web of social, economic, cultural, and gendered determinants. Pre-depature medical screening ensures that only those deemed physically fit can legally enter the country as migrant domestic workers; however, occupational stressors, as well as exposure to exploitation and abuse threaten to erode their mental and physical well-being [36]. The nature of the demanding work where individuals live and work in the private residence of their employer coupled with low socioeconomic status can contribute to stress and its attendant conditions related to lack of control over the workplace as well as limited access to determinants of health including social support, sufficient economic resources, and access to health care [37–40]. Indeed, little is known in Malaysia about foreign domestic workers’ health care utilization, self-care techniques, and access to other determinants of health such as nutrition, social support, adequate housing, among others. While working abroad, migrant domestic workers may be vulnerable to physical, psychological, economic, and sexual abuse as well as to other occupational exposures from cooking and cleaning materials [39, 41, 42].

## Theoretical framework

To examine these issues, we employ a dynamic multi-scalar lens that attends to intersectionality and centres the body as the site of human experience. Within this theoretical frame, the dynamic interpenetrating interactions amongst macro-to micro-level phenomena situate individuals and groups of individuals within social locations that configure exposure to power, privilege, and oppression, and that engender physical and social wellbeing, but can also and offer possibilities for agency [43, 44].

At a macro-level, neoliberal globalization and the structure of global political economy produces, through the racialized and gendered global division of labour, de-valued, flexible, and mobile subjects who are allocated to the lowest echelons of the labour market in migrant-receiving countries [44]. These macro-level processes bleed into meso-level phenomena. The social organization of the labour market, which both informs and is reinforced by gender ideologies and constructs of racialized status, is interwoven with the realm of national to community-level policies, discourses, and institutions that structure migration and border controls, access to health and social services, and labour market policies and practices, which reflect contemporary neoliberalism. Furthermore, ethnocultural context and socio-political environment underpin how individuals make sense of, and situate themselves, in the world. All of these multi-scalar interactions collapse into the proximate, micro-level scale of family, household, and ultimately, the body as the site of apprehension of the world where the whole of one’s environmental context is experienced, embodied, interpreted, expressed—and sometimes resisted—somatically, cognitively, and verbally [44, 45).

Overall, as Yeoh, Wee, and Goh note, focus on the corporeal in scalar analysis shifts our focus of migration from the economic to socio-political context and further allows us to highlight the pathways through which global processes get under the skin and impact health and wellbeing [46]. The precarity of labour and migration status further evidenced by restrictions in both mobility and claims-making, constant surveillance and self-surveillance due to the paucity of privacy, lack of control over the pace and conduct of work tasks, worry about family at home, limited access to determinants of health, and the mismatch between aspirations and imagined possibilities, and reality contribute to ongoing chronic stress and its sequelae, some of which defies nosological categorization, that can be understood as the embodiment of inequality [45, 47–50].

These complexities demand an attention to intersectionality. “As the sinew connecting identity, social structure and the materiality of social location, intersectional status—constituted by the historically and socio-politically constructed axes of social differentiation that structures access to social and material resources and power. Consequently, the intersectionality of various axes of social differentiation has implications for health, wellbeing, and social relations, and helps to illuminate health disparities and other forms of inequalities” [51]. The intersections of the dynamic interactions amongst social markers (gender, socioeconomic class, racialized and migration status, sexuality, dis/ability, *inter alia*) expose individuals and communities to various oppressions and privileges [50, 52–54]. The mutual constitution of gender, migration status, socioeconomic class, age, nationality, and ethnicity, among other axes of social differentiation, serve to structure and constrain health, which can be mediated by various coping strategies. Thus, within this framework the lives of migrant domestic workers are contextualized by sets of social relations that link the personal, communal, societal, and global.

## Methodology and methods

To examine the health and wellbeing of Filipino and Indonesian migrant domestic workers from their own emic perspective in Malaysia in the context of globalization, nationalism, and migration management policies, we conducted a multi-method study in Kuala Lumpur from 2008 to 2010.

### Positionality of team

Intersectionality is also a consideration when we reflect on the positionality of team members, the core group of which were Canadian and Malaysian women scholars of disparate academic rank and diverse ethnicities, class backgrounds, and religions. The first author, Spitzer, a white-presenting cis-gendered Euro-Canadian woman researcher, migrant, and former domestic worker, who, at the time of study, worked with Filipino migrant care workers in Canada for more than a decade. Thambiah is a Malaysian academic of Indian descent and a social anthropologist who works with marginalized communities in Malaysia. She has supervised graduate students working on migrant workers and they have supported the research in data collection and transcription work. Wong is a Malaysian academic of Chinese descent and a public health expert focussing on social determinants of health. She has researched, engaged in, and supervised interventions on gender-based violence and the health of rural-urban migrants. The core team was joined by policymakers and civil servants, of diverse ethnicities, engaged in advance study who provided input regarding policy, data collection and analysis, a Canadian racialized migrant woman research coordinator as well as Malaysian and Indonesian graduate research assistants.

Based on prior experience, we made strategic decisions about who might be best suited to conduct interviews. Data collection was primarily carried out by the bi-lingual, bi-cultural graduate students. In Malaysia, white privilege, the long-enduring residue of colonialism, means that often it is easier for a foreign researcher to gain access to policymakers and those in private enterprises. Thus, Spitzer, who assisted with some interviews of migrant domestic workers, was responsible for conducting the key informant interviews with policymakers, recruitment agency, medical screening clinic representatives, and non-governmental organizations (NGOs). Importantly, we engaged in group data analysis wherein members of the research team, student research assistants and research coordinator, and our policymaker and civil servant partners collaboratively reviewed the data from our myriad standpoints; this process helped solidify our analysis and strengthened confidence in our interpretations.

Our data collection involved triangulating multiple data sources (documents, interview data, and survey responses) to highlight the complexity of the issues at hand and to enhance the validity of the data by situating it within a broader context. Beginning with the documentary analysis, a team member examined government documents and websites to provide us with a depiction of the policy landscape that structures the lives of migrant domestic workers and their employers in Malaysia. Informed by the literature, interviews with NGO representatives, and team members’ knowledge from previous work with migrant domestic workers, we drafted a semi-structured interview guide and survey questionnaire that was subsequently translated into Bahasa Indonesia. Materials were not rendered into Tagalog as the Filipino migrant workers preferred to use English, the language in which they functioned in Malaysia, in their interactions with the research team. Investigators conducted training sessions with the research assistants, primarily graduate assistants at the University of Malaya, to ensure consistency in data collection. Interviews were recorded, transcribed verbatim, and where necessary, translated into English. Ethics approvals for primary data collection were obtained from the University of Ottawa and Ministry of Health, Government of Malaysia. Snowball sampling was used to recruit participants facilitated by local NGOs, foreign government representatives, and the personal networks of the research assistants. Interviews were held in a place agreed to by the informants and conducted in Bahasa Indonesia or English as per the interviewee’s choice. Prior to the commencement of the interview, participants were apprised of their rights as research participants as outlined in our consent forms.

In total, we interviewed 30 Indonesian and 24 Filipino migrant domestic workers. Approximately 33% of our Indonesian sample was married, and 13% were widowed compared to the Filipino informants, 18.5% of whom were married and 2% widowed. Two-thirds of the interviewees had children: of those 58.3% had one to three children, 8.3% had four, while 8.4% bore between five to seven children. Just over half of the respondents were from urban areas in their homeland. There were stark educational differences between the two groups of respondents: 37% of the Indonesian sample compared to 6.5% of the Filipino sample reported attending school for a maximum of one to six years. A quarter of the interviewees from the Philippines obtained some university training, 5.8% had been awarded graduate degrees. In comparison, none of the Indonesian research participants were educated beyond secondary school. Before migrating to Malaysia, over 70% of the informants were employed—most commonly as factory workers, farmers, or as vendors. Eight informants worked in other countries before moving to Malaysia; three Indonesian women worked in Brunei, Saudi Arabia, and Singapore respectively while two Filipino women were employed in Singapore, and one each worked in Saudi Arabia, Lebanon, and Hong Kong. Nearly 30% of our informants worked in Malaysia for more than 10 years; 22% lived in the country for five to ten years, and an equivalent number were employed for two to five years. Eleven percent resided in Malaysia for one to two years and the remaining 15% worked in Malaysia for under one year. In general, the Filipino research participants lived in Malaysia longer than workers in our Indonesian sample. Forty-seven percent of our interviewees worked for a single employer during their time in Malaysia.

In addition to talking to migrant domestic workers, we interviewed five representatives from civil society organizations, three government representatives and four individuals from the private sector involved in labour importation and health screening of migrant domestic workers. Information garnered from these sessions provided the social, economic and policy context within which we have situated this study.

Qualitative data were reviewed and refined in a face-to-face meeting with team members who devised a basic coding framework that emerged from the interview texts. Coding was facilitated by use of the computer software package, NVivo 7 and survey responses on demography were analyzed using SPSS.

## Results

### Departure and the stress of leaving home

Unsurprisingly given the socio-political context in which they were situated, three quarters of our informants cited economic difficulties as their primary reason for leaving their homeland to work in Malaysian homes. Sixty-one percent emphasized that their labour trajectory was undertaken to aid family members primarily with subsistence, education, health, and business. Just over 10% joined family members in the country and an additional 5% each specified that the global economic crisis and securing money for specific business ventures were their primary motivators for migration. Additional reasons for migration shared included martial problems, joining other friends in Malaysia, wanting independence, and searching for more work and life experience.

The information they had about Malaysia prior to their departure was spotty and conflicting. Many were aware of highly publicized abuse cases involving migrant domestic workers, yet they also heard that the country was peaceful and safe. Importantly, most had heard of others who had succeeded in meeting their financial goals by working as domestic workers in Malaysia. Limited in their ability to access labour rights, residency, and social support, our interlocutors often turned to individual strategies to help them cope with their working and living conditions. For example, respondents often spoke of the importance of prayer in helping them handle migratory stress. Praying for a good employer whom they could please also helped both to mitigate their fears of moving into a home filled with strangers in an unknown country, and to dispel any lingering media image they may have had of abused migrant domestic workers in Malaysia. Halma^3^, a middle-aged Indonesian widow with five children, summed up the sentiments of many other migrant domestic workers: *“I imagined it like this. If my luck is like the husk, I’ll float, if my luck is like the stone, I’ll sink.”*

Despite the financial hardship, the decision to work abroad was not easy to make. A married Sumatran woman in her mid 30s, Ani had to negotiate and persuade her father who disagreed with her desire to work abroad. Linda, an Indonesian mother of seven, simply disobeyed her husband, while Candy’s husband compelled this Filipina mother of three to leave and take a job overseas. On the other hand, Irda, an Indonesian in her early 20s, told acquaintances she was moving to Medan on the island of Sumatra in Indonesia instead of Malaysia while Maya, also from Indonesia, refrained from informing anyone about her departure. The Filipino respondents noted that migrant domestic workers were viewed respectfully in the Philippines for their industriousness and support for their families. Indonesian informants, however, reported more diversity of attitudes towards migrant domestic workers in their country; hence, some opted for a surreptitious departure. Seventy-two percent of the Indonesian and 81% of Filipino respondents remarked that there were many women and men in their home communities who had gone to work overseas, thus normalizing the option of labour migration. Halma offered another salient adage: *“It rains gold in someone else’s country; it rains stones in one’s own country.”* Being away from home and family especially for those who have left home for the first time can be very stressful.

### Migration process and cost: influence on well being

Respondents were asked why they selected Malaysia as a destination country. Three women indicated that they opted for Malaysia because their low levels of education would have made them ineligible to enter some other countries. Nine informants said that the recruitment agency directed them to this country offering no other option. For many, Malaysia was geographically close—and for the Indonesian respondents, culturally, linguistically, and religiously similar—to their homelands that they deemed working in Malaysia a less frightening prospect than traveling further afield. Eight informants came to Malaysia through chain migration where a sibling or other relation arranged for an employer to sponsor them.

Working abroad requires an initial financial outlay for costs of training, travel documents, medical screening, transportation, and other fees. Thirty-six respondents relied on recruitment agencies to finance their trip to Malaysia. Fifteen had their expenses covered by their employers, while six workers were aided by family members, and five paid their own way. Those who received assistance from recruitment agencies or their employers sustained salary deductions for three to seven months to reimburse their sponsor. Jen recalled her trip from Indonesia five years ago:

*…The expenditure of the journey to Malaysia. The first, from the village to Jakarta, my old folk were given 1 million rupiah for the expenditure for the time in Jakarta and then to Malaysia. After that in Malaysia the agent is responsible for everything. Then I was sent to the employer, but for five and a half months I was without pay because of the deductions for the agency.*

It is clear that the first few months upon arrival, they were without any income and this caused stress if family members requested funds unaware that migrant domestic workers had to wait a few months before being paid.

### Employment conditions and well being

Employers of migrant domestic workers are required to provide suitable accommodation and meals free of charge and are also responsible for the worker’s medical expenses in the event of illness or injury. Furthermore, special protection is afforded to Muslim workers in terms of their rights to religious practices, which remain paramount and as such that they cannot be directed to undertake activities contrary to Islam or that would interfere with religious observances.

Some of the unique features of paid domestic labour include the private nature of the workplace, the close proximity of one’s employer and the lack of separation of work and leisure [27, 55, 56]. Previous studies suggest that living with one’s employer is the least desired option for migrant domestic workers who are employed in countries where live-out migrant domestic workers may be legally employed [56]. In this study, informants reported that they often shared rooms with their primary care recipients, generally children; however, the majority asserted that they had sufficient privacy. Rina, a widowed Indonesian and mother of four shared how lack of privacy to have her own room interrupted with religious obligations.

*The children are naughty, really. The little one sleeps with me, too. That has been my set-back. I wish...wish I told my employer so that I can carry out the prayers of five times. For that I haven’t been given the time.. .. What else when there’s so many children, can we concentrate if we want to pray? … If I cannot, yeah, already cannot, I’m sure to go to hell.*

Most of our informants declared they had positive relationships with care recipients. While a small minority preferred more formal relations with their employers, most migrant domestic workers favoured warm relations and indeed many felt they were treated like a member of the family. Communication was seen as key to developing and maintaining good rapport with employers; however, the incipient power relations that underpin the social hierarchies that are replicated in the household are evident regardless of the quality of employer-domestic worker relationships. Rina, whose religious observances have been disrupted by her young charges, feared discussing the situation with her employer as she was unsure whether her employer would sympathize with her predicament and would be willing to take action to remedy it. Lhet, a Filipino domestic worker who has resided in Malaysia for more than a decade said of her employer who likes to treat her as a friend: *“She wants you to smile even if you are tired, angry. You must smile.”*

The social hierarchy is further evidenced by responses to our queries regarding social interactions with other household members. For instance, 46% of our informants shared meals and leisure activities with their employers—although a small number would have preferred not to do so. Thirteen percent would like to engage in these activities with their employers, but do not while 39% did not share meals and had no expectations to do so themselves.

Sixty-one percent of our respondents stated that they are constrained in their use of space when their employers were home, although some reported receiving warnings against using certain space or items such as the telephone when the employer was not home. The close proximity to their employers however means that they either feel under surveillance or place themselves under further self-surveillance. Linda, a Christian Indonesian mother of seven said:

*When the employer is here, I too don’t watch TV. If the children were watching cartoons, yeah, I go along. I’m afraid, if…didn’t say, but what if [the employer thinks], ‘Oh like that’s what she does at home. Watching TV all the time ya right?’ Aa…because the employer doesn’t look for the good of the maid, but always the faults. I have observed my employer, always regarding my work, the part she takes note of are my faults.*

The power of observation also appeared to be key to maintaining their jobs as respondents described watching employers closely to ensure they were following instructions and anticipating their needs. Informants were generally assigned similar duties including housecleaning, laundry, childcare and/or eldercare, cooking, cleaning cars and gardening. Interestingly, pet care has also become increasingly common, and informants cared for a host of animals—fish, tortoises, rabbits, and hamsters as well as cats and dogs. The days’ activities are generally structured around the comings and goings of household members. Rosanda, a young, single Filipino woman, describes a typical day:

*I get up in the morning about 5:30, then I take all the clothes to put in the washing machine, then cooking for my Mem’s lunch to bring to the office, then… after that I am going outside cleaning the car, then mopping the floor there, and then after I’m making breakfast for my boss then after making breakfast, I am going out again and wash the other car outside. Then after doing work in the outside, I’m having a breakfast, then washing the hand wash clothes then after that start cleaning already. Then after 11:00, I cook for lunch for the kid, then… after that [I] continue cleaning, then after cleaning ironing, then after ironing preparing for dinner and then until nine. Because my Mem also she came back late. . So, since 10:00 then I can go (sic.) inside my room.*

Since the completion of our data collection, the Government of Malaysia has mandated a single day of rest for migrant domestic workers to be determined by the employer [28, 29]. At the time we conducted interviews, migrant domestic workers were to be allotted one rest day per week described on the Department of Immigration website as a continuous period not exceeding 24 h—although in the Malay version, it is defined as “sufficient rest including at least 8 hours of sleep per day” [57]. Of our sample, 45% already received leave each week although 22% reportedly never received a day off. Among this group, Candy, a Filipino mother in her late 30s, had not had a day off in four years while for her compatriot, Lhet, it has been two years. Over 11% were granted one day off per month while 8.5% were granted two per month. A few other arrangements were uncovered; 3% of the sample were given three days per month; another 3% were off work for two days every two months; an equivalent number had three to five rest days every three months or were just granted rest days on an irregular basis. For some informants, the definition of *rest day* was called into question. For Elsa, a 40-year-old Filipino domestic worker who has lived in Malaysia for more than 12 years, her rest day consisted of two hours every Sunday. Peni, a policymaker, spoke about another common issue that emerges from negotiating the boundaries between employer-employee and friendship.

*I have friends that have maids. … This one is, I think, a good employer; like if my friend sends the family go for vacation. So, this maid, if she wants, if she wants to go with them for vacation then they’re allowed. If not, ok we want to go on vacation this day, you must stay home. Then she can go wherever she want. That one is the one side of story—the good side of story, the other side then you need to go with the family. You don’t, actually even the family go for vacation, for holiday, the maid is still doing the work. Taking care of the children and then the family go for shopping, picking up the [groceries].*

Like Peni’s friend, some employers are cognizant of the difficulty of extracting oneself from an employee or employer roles resulting in the enactment of these roles even on holidays. Twenty-percent of respondents also received overtime pay in recognition of workload increases. The employment condition and work environment, therefore, is inextricably related to the well-being of the domestic worker.

### The denunciation of desires and dreams: influence on well being

In recent decades, the demand for migrant domestic workers, particularly women, has burgeoned. Gender ideologies modulated by culture, class, religion, place, and other factors, are embedded in social interactions, expectations, decisions, and structures that shape women’s gender roles and the performance of certain gendered dispositions (e.g., docility, pleasant demeanour) reflect the desires of recruitment agencies and employers. Many of our informants learned to abnegate their desires and dreams to allow male and/or younger siblings to prosper. As one of seven children in a poor household, Cristy, a Filipino mother of one, shared that her family opted to only educate her brothers. As a girl in the Philippines, Kathy had hoped to become an engineer, but instead she was set to work to put her younger sisters through school. Now that they are married, as the eldest sister she is expected to continue to sacrifice her own aspirations and provide ongoing support for her sister’s family that continues to be in need. To this end, she has been working in Malaysia since 1988 and, at the time of our interview, was in her mid 40s. Kathy, therefore, saw no chance to either study or marry and start a family of her own.

Some migrant domestic workers migrate to work to escape domestic violence and they continue to carry the pain of violence in their memory, which impacts their well-being while they are in Malaysia. Linda, a Christian Indonesian woman in her mid 40s, articulated how globalization, gender, work, and health are embodied in her lived experience:

*I wanted to be a defender of women, but because of the economic factor, I couldn’t. Because I see in Indonesia, we are, we women are given less attention, ya right? So, there are many women who are suffering because of their husbands. Those who came here too had problems from their husbands, right? So since last time, if I observe, I have been hurt by my husband. I asked God: “God don’t have anymore Indonesian women like me.” Always tortured by husbands. It’s not just bad, worse than bad. He drinks, womanizes, hits. [back to crying] That’s why for only three years I was married to him. How God, our government. I always pray, asking God: “God bless our government. Bless our country. So that we no longer need to go to Malaysia.”*

### Health status and access to health services

Overall, two thirds of both Indonesian and Filipino informants reported no change in their health status since moving to and residing in Malaysia. Eighteen percent of Indonesian and 22% of Filipino migrant domestic workers claimed their health was better in Malaysia than in their home countries while 14% of Indonesian and 11% of Filipino workers felt that their health status had declined. Headaches were the most reported complaints. Hypertension and menstrual problems were the next most common conditions while some mentioned having diarrhea and others had problems with their eyesight.

Except for two informants who reported having difficulty accessing health services because their employers would not allow them to leave the workplace to seek help; the rest of the respondents were satisfied both with their access to, and with the quality of, health care in Malaysia. Employers played a vital role in helping workers navigate the health care system, ensuring they received appropriate and timely care. According to government regulations at the time, employers were required to cover the costs of health care services for their employees unless they are still under the auspices of the recruitment agency who were then responsible for those expenses. Approximately half of respondents had health insurance coverage paid for by their employers. Among the half who did not receive health insurance coverage, 17 reported that their employers still paid or reimbursed their health bills. Twelve respondents paid for health services themselves—a few stating that they were “too shy” to approach their employer to reimburse their health expenditures.

Women also recounted their interactions with FOMEMA the organization that manages the health screening of migrant domestic workers in Malaysia. Foreign workers, depending upon their occupational status, are as previously mentioned, required to undergo annual and then more infrequent health examination for a variety of primarily infectious or chronic conditions that might impede the worker’s ability to complete her duties. The Department of Immigration receive the judgment of physicians who are in essence agents of FOMEMA as to whether the individual is deemed fit or unfit. Respondents were generally satisfied with the health examination process although there were some disparities in the way in which the results were distributed; some say they were sent directly to their employers; others received them directly. Although there were also differences as to the number and type of tests that they underwent with FOMEMA, the comparisons between the medical examination screening they had in their home country prior to departure generally found favour with the Malaysian system which was viewed as more efficient, orderly, and respectful. Indonesian informants in particular were more satisfied with the Malaysian medical examination as compared to the medical examination they underwent in their home country; however, a small number of Filipino informants felt that the examinations they underwent in their homeland were complete and more compassionate. For some, the thoroughness of the examinations, both pre-departure and in Malaysia, meant that patients were asked to disrobe, enhancing their stress and hesitancy to undergo subsequent routine exams. Some respondents shared how these examinations were unpleasant experiences for them. For example, three Filipino respondents reported they were asked to fully undress for their pre-departure medical examination in the Philippines. Kathy queried the physician’s request that she take all her clothes off and bend down; the doctor responded, “This is a medical check-up. This is the one you need.” Candy noted that medical examination protocols appear to have changed when she returned to Malaysia for a second time.

*In the Philippines, [the physician said] all the way take out, lah. It’s like naked already because they are going to check the whole body. … First time I came here, I never experience about that, must be naked.. . but now when I second time came here already, the FOMEMA ask me to be naked. Yeah, I’m naked together, the doctor is there…together with the nurse.*

### Promoting health and well-being

Being surrounded by people who make one happy, trying to not think too much, practicing patience, and refraining from anger or unpleasant thoughts were amongst the most cited contributions to good health status. In addition, one had to attend to the body through exercise, proper rest, healthy foods, drinking clean water, and eating and sleeping in accordance with nature’s daily rhythms. Some of the Indonesian informants also maintained their health by consuming *jamu*, traditional medicine of Indonesia. The converse of these activities contributes to poor health including most saliently overwork, over-thinking, and stress, particularly when one is pining for one’s family. Kathy, a middle-aged domestic worker from the Philippines, described how she used self-care activities to distract her from missing her family:

*[I am] drinking a lot of water. Little bit exercise. But now I exercise every morning. Really exercise because washing the car is one exercise, mopping the floor. Not really exercise that one, right? And I am not thinking about problem, but you cannot also avoid the problem, right, if you thinking every time the problem, you so fast become old. . Every morning I wake up, I sing the praising song for distraction. . If you have a lot of problems, if you thinking because if you’re mind thinking, your mind [is] not feeling well. That’s why I never think. Yeah, I never.*

While these modalities of self-care are individualized methods of health promotion, interlocuters also strove to leverage social support, another vital determinant of health that can in fact be highly problematic to access for migrant domestic workers due to their isolation in private households [37, 54]. The Filipino informants who, at the time of data collection, had more regular days off than their Indonesian counterparts become involved in a variety of community organizations, from Philippine associations, church groups, sports clubs, to arts and crafts organizations and church choirs. For many informants who had limited social interaction, employers became the primary source of social support followed by family at home and for those who have them, family members in Malaysia. Five informants said they turned to the agents who recruited them for assistance: one utilized formal support services. Most respondents felt they had sufficient social support even though they did not often try to mobilize their support networks. Seventeen participants, however, described how they had to cope with a paucity of social support. For example, Liza, an Indonesian mother of two, relied on her own inner strength. She shared that: *“No one supporting; it’s from my own thoughts.”* Many drew strength from prayer and spiritual practice for solace and support. Candy, a Filipino mother of three whose husband compelled her to become a migrant domestic worker said:

*If I have a problem, I never think about that lah. Just… because I’m here in Malaysia, of course a lot of friends but if for example, you have a problem also, sometimes the friends cannot help you also, lah. So… I always think positive. I pray to God, I’m here in Malaysia to work. Then, I’ll pray to God that he doesn’t give me an illness lah. Hor? I don’t want, I don’t want to become sick here in Malaysia because I’m working here. Not only for myself, but for my family.*

Halma, a 56-year-old widow who had worked in Malaysia for 15 years, shared her lament for her husband and how she copes:

*[I] think about… why did you leave all your children to me? Like that lah... Why did you go so soon, like that? [Interviewer: So to reduce the stress, what does ibu*^4^*do? Or even prevent, so as not to be stressed what does ibu do?] I get up in the middle of the night, pray. Pray, asking God for patience and strength in my heart. Get up in the middle of night so its calmer.*

Prayer was mentioned by many informants to give them a sense of calm and it was used as a self-care strategy to help them relax and cope with stress.

## Discussion

Contemporary neoliberal globalization has restructured economies and facilitated the circulation of people and capital [1]. Importantly, the dynamic interactions and the overlapping of various markers of social differentiation are implicated in who, how, and why certain sectors of the global population are mobilized as temporary migrants, how migrant workers are treated, and how their circumstances, underpinned by globalization leads to their precarious existence impacting their well-being and health. Neo-liberal state discourses and mechanisms propagate migrating for work as a poverty alleviation strategy— an emerging abandonement of the state in providing a liveable life, while distancing its responsibility towards its citizens’ wellbeing and health. In addition, gendered, cultural, and religious values and ideologies underscore filial duty and sacrifice for the family and in some instances lack of opportunities in the home country for women fleeing gender-based violence, helping to further impel migration, which coincides with the increasing demand for feminized domestic labour. Furthermore, historical, linguistic, cultural, and political connections and state apparatuses have helped to create Malaysia as a popular destination country for Indonesian and Filipino migrant domestic workers.

The health and wellbeing of Indonesian and Filipino migrant domestic workers in Malaysia is grounded in their social location—the dynamic and cumulative product of multi-scalar processes and intersectionality which configures access to determinants of health. The construction of Indonesian and Filipino women as suitably docile gendered racialized bodies who can be readily inserted into the flows of Malaysia-bound migrant workers and then deposited in private households situates them in a novel social landscape. Here, they may be economically unstable (especially when recruitment agency fees consume much of their income), which in itself may not be an unfamiliar circumstance in their home countries, but in Malaysia, they are separated from homeland, family, and friends, and must contend with precarious work and migration statuses, the collapse of homelife into the world of work, with little control over either. Low socioeconomic status, a paucity of social support, their precarious migration status and restrictive gender roles constrain agency. These factors along with immobility are among the determinants that directly, as the result of poverty or noxious environmental exposures, or indirectly through long-term stress, erode health and wellbeing over the long term. As the multi-scalar effects of neoliberal globalization coalesce and are responded to by individual bodies, some manifest as identifiable diseases or conditions, others as inchoate symptoms (e.g., headaches), all of which can be regarded as the embodiment of inequality. Moreover, these processes and their effects are not uniformly distributed. Filipino and Indonesian migrant domestic workers at the time of our research were generally accorded different social and economic statuses reinforced by state to state arrangements and discursive constructs of gendered ethnicized figures—Filipino migrant workers as economic heroes with greater sophistication and education and Indonesian migrant workers as naïve and docile subjects who required greater protection from the outside world. These tropes impacted employers’ attitudes regarding employees and whether to allow them to avail themselves of their legislated rest days with employers of Indonesian workers more likely to disallow workers to leave the household in the name of keeping them from harm. Those Filipino workers who had more opportunities to leave their employers’ homes than their Indonesian counterparts became involved with local cultural and religious organizations, which helped to moderate their stress levels. The inability to claim rest days increases stress levels and reduces access to social support. Notably, social support was identified by many of our interviewees as being virtually synonymous with good health and wellbeing and with minor exceptions many experienced a profound lack thereof. In addition to some disparities between workers of different national origins, employers’ socioeconomic status, household configurations, and personalities further influence migrant domestic workers’ social location and access to health determinants.

While most of the Indonesian and Filipino migrant domestic workers we interviewed felt they had attained success and were generally satisfied with their decision to come to Malaysia, their lives were not without struggles. They remained constrained by the structure of migrant domestic work that continues to fail to recognize their labour as bona fide work and the private accommodations where they toiled as workplaces not covered by legal and social protections. Although many informants claimed to be treated like a family member by their employers, unlike other family members, they were sometimes constrained in their ability to use household items or space. Furthermore, where family members anticipate reciprocity in their relationships or are relatively free to come and go or invite visitors home, the same could not be said for migrant domestic workers. Indeed, employers who claim their migrant domestic worker as fictive k in are more apt to extract additional labour from them, thereby erasing the façade of being a true member of the family.

The migrant domestic workers we interviewed were generally satisfied with access to and the quality of healthcare services in Malaysia, despite concerns about the nature of the pre-departure medical examinations (reputedly demanded by Malaysian authorities) some had experienced. Moreover, the impact of migration on their self-reported health status was mixed, with two thirds of respondents noting no change. Importantly, this finding does not mean that they experienced good health and wellbeing as an outcome of their migration but that they were required to be healthy to migrate for work in the first place and therefore most respondents noted no change in their health status. Upon migrating the precariousness of their lives as migrant domestic workers and the separation from family and friends impacted their well-being. Both the Philippines and Indonesia have experienced ongoing economic and social crises for years [1], thus, migrant domestic workers may well have traded precarious lives in their home countries for precarious lives in Malaysia, with all the attendant pressures and health effects associated with low waged, low status employment. As migrant domestic workers primary focus was on remitting funds to their families, worries about not providing enough money, concerns about family members from whom they were separated, and the paucity of social support threatened to generate poor overall wellbeing.

Although migrant domestic workers sustained various forms of deprivation including long working hours, infrequent rest periods, lack of control over work, lack of privacy, surveillance by employers, they were often able to withstand their impacts by focusing on their goals and the anticipation of their return home. Importantly, individual self-care and spiritual practices served as vital ballast against the pressures they faced. The strain of maintaining that stance over a prolonged period, however, can result in an eventual erosion of health status over the long-term. The routine medical screening that migrant domestic workers undergo to take up and maintain their posts in Malaysia evaluate physical health, the presence or absence of quantifiable disease; however, our interlocutors grounded definitions of health in the context of wellbeing, situated within the context of their lives, communities, and locations as women migrant domestic workers separated from family. With limited access to social determinants of health, including economic wellbeing and labour rights, the migrant domestic workers we met primarily employed individualized self-care practices that may contribute to physical health and offer some temporary solace; however, longer term social and emotional wellbeing as an effect of the dynamic interactions of multi-scalar processes and intersectional status, and individual embodied and communal responses were acknowledged but less often acted upon.

## Conclusion

Structural inequities and the mobilization of gendered values that reward self-abnegation underpin migration as a development strategy. While individual self-care practices were importantly used to ameliorate the hardships of their work and family separation, these efforts cannot remedy overarching, long-term harms nor redress structural inequities wrought by neoliberal globalization. Raising salaries, social and labour protections that would acknowledge domestic labour as work and households that hire domestic workers as workplaces, and assurances that legislated rest days are diligently implemented, would help contribute to better physical and mental health of migrant domestic workers in the short and medium term. Improvements in the long-term health and wellbeing of Indonesian and Filipino migrant domestic workers in Malaysia can only be wrought through their attainment of adequate social determinants of health, which requires a critical interrogation of the migration as development paradigm and greater attention to the far-reaching effects on neoliberal globalization. Importantly, the various faces of neoliberal globalization intersecting with migration and health have redefined the role of the state and society, where the state through the ‘migrating out of poverty’ model of development shifted its role to society and private actors. In the analysis of neoliberal globalization and health, there is a great deal of focus on only healthy bodies being permitted to migrate for work– as required migrant domestic workers undergo dual health screening, pre-departure and before a work permit is issued. Neo-liberal policy instruments such as privatization, marketisation and commercialization of migrant workers have led to both host (freeing their women from reproductive labour to participate in the labour force) and home countries benefitting (the state shifting the responsibility of moving its society out of poverty and bringing about development through remittances), but all these reputed wins come at the expense of the migrant domestic workers well-being.

## Data Availability

The datasets generated during and/or analysed during the current study are not publicly available as storing data in public respositories was not an option at time of data collection. Data may however be available from the authors upon reasonable request and with permission of the University of Ottawa, Universiti Malaya, and National Institute for Health Behavioural Research.

## Footnotes

1 Please note that we are describing the political and economic landscape at the time of our study from 2008 to 2010.

2 The minimum salary of an Indonesian domestic worker in 2019 was RM1,000 (US $250) per month whereas a Filipino domestic worker would expect to be paid a minimum of RM1,600 per month (US$400) a month. Minumum salaries for Filipino domestic workers has been raised to RM1,800 during the COVID-19 pandemic. Currently, the minimum monthly salary for Indonesian domestic workers is RM1,500 [58].

3 All names are pseudonyms.

4 Polite address for older woman.

## List of Abbreviations

CEDAW: Convention on the Elimination of All Forms of Discrimination
FOMEMA: Foreign Workers Medical Examination Agency
ILO: International Labour Organization
MOU: Memorandum of Understanding
NGO: Non-Governmental Organization
